# Supporting Equitable Aged Care Access: Feasibility and Acceptability Pilot Study of a Paeārahi-Facilitated interRAI Self-Assessment Model for Indigenous Elders

**DOI:** 10.1177/08404704251369754

**Published:** 2025-10-09

**Authors:** Joanna F. Hikaka, Mariana Foxcroft, Karyn Foley, Sally Aydon, Robinson J. Spencer, Brigette Meehan

**Affiliations:** 1 1415 The University of Auckland, Auckland, New Zealand; 2 Health New Zealand, Wellington, New Zealand; 3 Health New Zealand, Hastings, New Zealand

## Abstract

An interRAI assessment is required for older people in Aotearoa New Zealand (NZ) to access public aged care services. Paeārahi (Māori health navigators) provide culturally-appropriate, connected healthcare. We investigated the feasibility and acceptability of paeārahi-facilitated Check Up Self Report (CU-SR) completion with older Māori in NZ. Prospective non-randomised, non-comparator intervention study in one NZ health practice with eligible participants (Māori, 55 years or older, community-dwelling, not known to require formal needs assessment). Predefined feasibility and acceptability outcomes were reported using descriptive statistics and thematic analysis. Participants (n = 50, mean 65.3 years, 66% female) felt most CU-SR items were acceptable. Paeārahi-facilitated assessment and care planning were generally acceptable and feasible to undertake and perceived to improve healthcare access. Paeārahi-facilitated CU-SR assessment and care planning is a scalable model utilising a culturally appropriate, non-regulated Indigenous health workforce and an internationally validated assessment with the potential to identify unmet need and address inequities in aged care access.

## Introduction

In Aotearoa New Zealand (NZ), completion of a comprehensive interRAI assessment by a trained interRAI assessor is required to access publicly funded aged-related care.^1^ In contrast, the briefer interRAI Check-Up Self Report (CU-SR)^2^ is used to gain a person’s direct perspective on their health and well-being, without requiring a trained assessor. Internationally, CU-SR self-completion demonstrates feasibility and acceptability to a cross-section of community-residing adults, with a high degree of agreement between the self-report and subsequent assessment by clinical assessors.^3^ The CU-SR has also been validated showing high levels of agreement between ‘lay interviewers’ administering the CU-SR and trained clinical assessors administering the clinical version of the interRAI Check-Up.^4^ The CU-SR has not been used in NZ previously or shown to be appropriate for use with Indigenous peoples.

Access to publicly funded home care services varies by ethnicity in NZ.^5,6^ Māori (Indigenous people of NZ ;18% of population; life expectancy approximately 7-8 years younger than non-Māori) are less likely to receive publicly funded care than non-Māori,^6^ and have inequitable access to interRAI assessment.^5^ It is unclear if this finding is due to individual and family preference, reduced access to assessment and care services, or a combination of both.^7^

In other settings, paeārahi (Māori health navigators) successfully provide wrap-around support for individuals and family collectives to manage chronic conditions and well-being, with the potential healthcare access and to improve integration across the health system.^8,9^ Such wrap-around is a central tenet in Māori care provision– resulting in a family-centred, strength-based, shared assessment and care experience. Paeārahi have knowledge of the Māori language and cultural practices, strong networks and connections, and utilise these to work with clients and their wider family to identify their needs and aspirations, support participation in core sectors such as housing, and employment, and coordinate access to primary care and specialist services.^10^ In the NZ context, it is an efficient, cost-effective and scalable model that does not require large infrastructure costs, and is translatable to a range of existing health settings.^8,9^ We aimed to test the feasibility and acceptability of the paeārahi-facilitated CU-SR in older Māori in one region of NZ.

## Methods

We conducted a prospective non-randomised, non-comparator intervention study with pre- and post-intervention measurements of predefined outcomes [prospective registration: Australia and New Zealand Clinical Trial Registry (ACTRN: ACTRN12623001253695p); ethics approval: New Zealand Health and Disability Ethics Committee (Reference: 18971).

### CU-SR Cultural Adaption

The CU-SR was developed internationally with robust testing and validation.^3,11,12^ Based on consultation, we made additions to the study version of CU-SR to include cultural values beside each item, for example manaakitanga (showing respect, generosity and care).^13^ Item order did not change and all original wording was retained with te reo Māori (Māori language) words added alongside. Based on consultation, the CU-SR was administered using a physical version (39 pages).

### Paeārahi Training

Paeārahi, with no prior interRAI assessment experience, underwent bespoke training co-designed with paeārahi. Training involved on-line learning self-directed modules followed by a 1-day in-person workshop style training delivered by interRAI educators. interRAI educators reviewed the first five assessments completed and were available for ongoing paeārahi support. Training focussed on the need to retain the self-report aspect of the assessment by asking the exact wording of CU-SR items, recording participants responses rather than others’ perception of the answer, use of interRAI software, item intent, and the Culturally Appropriate Assessment Model (CAAM; developed by drawing on Indigenous health models and relational practice^13^).^14^ The CAAM incorporates Indigenous Māori cultural values and practices, from initial contact through to follow-up post-assessment. Many CAAM values are inherent in the way paeārahi practiced. Needs Assessment and Service Coordination (NASC) clinicians provided training on their role and service and information about what needs and services may be triggered by interRAI assessment. Paeārahi shared their roles with NASC and educators. The lead researcher provided paeārahi with training in the research processes and study protocol.

### Population, Recruitment and Consent

A Māori-led health centre, whose employees include primary care physicians and paeārahi, was a partner in this research and participants were recruited by centre staff.

Inclusion criteria: Māori, 55-plus years old, community-dwelling, study practice patient, ability to understand written and/or spoken English. Exclusion criteria: referred for/previous interRAI assessment.

Health centre staff identified eligible patients and referred to paeārahi through the internal Patient Management System (PMS). Paeārahi contacted eligible patients to gauge interest, then met to discuss the research and obtain in-person consent.

### Intervention Delivery

The intervention consisted of CU-SR completion with participants either completing the written CU-SR themselves or with paeārahi support to read items and document answers. Paeārahi entered the assessment data into the interRAI Assessment Software (iAS) and used results to care planning, identifying actions to be taken, consulting centre staff as needed (as per their standard practice). The drafted care plan was reviewed by a NASC clinician from secondary care. Paeārahi documented actions and referrals in the iAS and PMS and arranged follow up.

### Culturally Appropriate Development and Implementation

We followed practices aligning with the He Pikinga Waiora Implementation Framework^15^ in the development of this intervention and research, cognisant of the intention to undertake culturally appropriate practices and inform future implementation. The key concepts from this model were: cultural centeredness, community engagement, integrated knowledge translation and systems thinking. This model informed ethical practices in the context of Māori-centred and led research with a focus on Māori leadership and shared decision-making with the Kaupapa Māori study practice. Paeārahi, employed by this practice and known to the community undertook research data collection in addition to delivering the intervention to increase the likelihood of participants trusting the research process and being willing to be involved.

## Outcome Measures

Demographic data and CUSR scores for scales, acceptability and feasibility outcomes were reported (Table 1).

**Table 1.** Outcome Measures, Data Source and Time Point for Collection

Outcome measure	Data collection method	Description	Collector	Data collection time points
*Acceptability*	Focus groups with participants, paeārahi, those involved in the intervention training, delivery and/or follow up health or social service provision. A semi-structured approach with topic guide (piloted among steering group) was used to facilitate focus group discussions. Focus groups were audio recorded and transcribed	Paeārahi selected up to five participants each, using convenience sampling, and asked via telephone if they and their family would like to participate in a focus group. The lead researcher contacted others to ask via email if they would like to participate a focus group. Focus groups were conducted either in a community location convenient to participants or via online videoconferencing. The lead researcher obtained written informed consent from all focus group participants immediately prior to facilitating in-person or online focus groups. Participants had been provided with information about the lead researcher prior to data collection and had the option to bring support people. Acceptability measures were co-developed during consultation meetings	Lead researcher (female, research fellow, PhD experienced in qualitative and health services research) facilitated focus groups	3-month post-intervention
Strengthening well-being				
Resulting services support independence and function				
Culturally appropriate assessment and process				
Improving the capability and capacity of the paeārahi workforce				
Assessment is fit-for-purpose				
Number of items which cause confusion or discomfort				
*Feasibility*	Study specific data collection form	-	Paeārahi	Immediately post-assessment
Time taken to complete assessment				
Number of visits to complete assessment				
Participant retention				
Time taken to travel to participant				
Time taken to follow-up post-assessment				
Assessment outputs are valid for individual and population-based reporting	Focus groups, as above		As above	3-month post-intervention
*Access to healthcare services*	interRAI self-assessment	-	Researcher	3-month post-intervention
Number of assessments completed				
Number and type of referrals	Patient Management System			
Number of referrals for further interRAI assessment	Patient Management System			
Number eligible for funded home care	Secondary care data service provision data		Secondary care data query	
Participant and family experiences	Focus groups, as above		As above	

Well-being was also measured at baseline using the modified Hua Oranga tool. ** McClintock KK, Mellsop GW, Kingi TKR. 2011. Development of a culturally attuned psychiatric outcome measure for an Indigenous population. Int J Cult Ment Health. 4(2):128-143

### Data Analysis

We used descriptive statistics to depict quantitative outcomes. C Cronbach’s alpha was used to measure internal consistency of Activities of Daily Living (ADL), Independent Activities of Daily Living (IADL) and mood scales using previously described cutoff points (alpha value ≥0.70 indicating acceptable reliability)^16^ and benchmarked against reliability performance of the CU-SR internationally (ADL = 0.89, IADL = 0.87, mood = 0.79).^3^

We analysed qualitative data by grouping under acceptability and feasibility with additional themes developed using general inductive analysis.^17^ The lead researcher coded the data, drafted themes, and presented findings to the steering group for consensus building and further contextualisation of findings.

### Steering Group

A steering group made up of interRAI staff, commissioners of aged care services, researchers, and primary and secondary care providers was established to support intervention and research design and implementation. Approximately 30% of the group was Māori. The steering group was established to increase the likelihood that the proposed intervention would be practical to implement in real-world settings and engage relevant stakeholders for future translation of research findings into practice, increasing the relevance of the research to the community or interest and to health systems planners and commissioners.

### Sample Size

We took a pragmatic approach to sample size, aiming for 50 completed assessments.

## Results

Fifty people were recruited from January to May 2024. The median participant age was 65.3 years (56-84 years), 66% were female and 98% were Māori. Demographic, clinical characteristics, and outcome scales are presented in Table 2. The IADL and mood scales, but not the ADL scale, demonstrated acceptable internal reliability (alpha scores 0.74, 0.77 and 0.37 respectively).

**Table 2.** Participant Demographic and Clinical Characteristics, and Outcome Scale Scores

Characteristic	Intervention participants (n = 50)
Median age (range)	65.3 years (56-84)
Female	66%
Ethnicity	
Māori	98%
European	2%
Residence	
Private residence	92%
Boarding	6%
Homeless (with or without shelter)	2%
Co-location	
Lives alone	28%
With spouse (+/− others)	38%
With child	20%
With others not listed	14%
Marital status	
Never married	34%
Married/defacto/civil union	32%
Widowed	12%
Separated or divorced	16%
Other	6%
Hua Oranga Domain	% rated domains as good/extremely good
Wairua (spiritual health)	86%
Tinana (physical health) good/extremely good	78%
Hinengaro (mental health) good/extremely good	86%
Whānau (family health) good/extremely good	94%

Characteristic	Intervention participants (n = 50)	
Scales		Trigger point
*Activities of Daily Living (ADL) Hierarchy Scale (0-6)*		Score of ≥3 indicative of moderate-severe impairment of ADLs.
Intact (0)	46	
Minimal Impairment (1-2)	2	
Moderate to severe (3+)	2	
Independent Activities of Daily Living (IADL) Capacity Hierarchy Scale (0 - 6)		Score of ≥3 indicative of moderate-severe impairment of IADLs.
0-2	48	
≥3	2	
Cognitive Performance Scale 2 (0 - 8)		Scores ≥2 indicative of potential early cognitive loss.
		Scores ≥3 indicative of a moderate to very severe cognitive impairment.
0-1	22	
2	23	
≥3	5	
DIVERT Scale (1 - 6)		The higher the score the greater the likelihood of ED presentation within six months of assessment. A score of 6 is indicative of highest risk.
0-3	47	
≥4	3	
Pain Scale (0 - 4)		Score of 2 indicates daily pain but not severe. Score of 3 or 4 indicates daily severe or excruciating pain.
0-1	31	
≥2	19	
Self-Rated Mood Scale (0-9)		A higher score suggests a greater likelihood of clinically significant depression or mood-related distress. A score ≥3 or higher is a strong indicator that the individual may require further assessment or intervention.
0-2	35	
≥3	15	

Fourteen people took part in three focus groups (n = 8 participants/family members, n = 6 paeārahi/health providers [providers]). The median age band for participants and family was 65-74 and 55-64 for providers. 81% of all focus group participants were Māori and 81% were female. Of the providers, 50% were registered health professionals, in the health sector and their current roles for a median of 15.5 years (3-30) and 2 years (0.5-10) respectively.

### Acceptability

Many participants reported the CU-SR as largely acceptable although they found the assessment repetitive and required a lot of reading. Some participants were unfamiliar with some diagnosis terms and found it difficult to complete without the support of paeārahi. Others wanted to discuss the questions with family members before responding. Several participants found the self-assessment confronting, especially when comparing their current well-being to previous good health. In contrast, those in good health reported completing it easily and quickly.

Participants felt the CU-SR provided a good level of information about themselves or those who were being assessed. Additionally, paeārahi reported benefits in relationship development through the assessment process, perceiving that this helped get a true sense of the person and what supports may be beneficial.

Some participants felt it was a ‘Western’ assessment, which adversely affected their approach to health provision

It’s a [Western] assessment so when you are completing that assessment, you look through a [Western] lens and think about what’s available in the [Western] system. So, you close your world off. – Provider

Inclusion of Māori language was important with one participant saying, ‘*Māori [words] are important, that’s our identity’*. However, many participants felt it was not necessary to incorporate Māori values alongside assessment items as they believed paeārahi already embedded these values in their approach. When asked whether the process upheld participant autonomy, participants acknowledged that their voices were heard but noted this still occurred within the constraints of a pre-defined assessment.

Those with experience of assessor-led assessment felt the number of items in the CU-SR may have been off-putting. Even though there are many fewer items than other interRAI assessments, they talked of how trained assessors completing other interRAI assessments have the flexibility to assess multiple items through conversation, reducing perceived discomfort or repetitiveness, which was not done with the CU-SR in this study.

### Feasibility

Introducing the paeārahi role to other health providers played a key role in implementing the service. During the pilot, paeārahi learned more about the roles of trained assessors and related services. At the same time, health providers in primary and secondary care gained a better understanding of the paeārahi role and its benefits

Participants felt that as long as paeārahi understood the intent of each question, they could rephrase them while still maintaining the assessment’s integrity and ensuring the older person’s response was captured. Without this flexibility, they believed it could harm the rapport built during the assessment

Paeārahi level of comfort undertaking the assessment grew over time and that supported completion, rather than self-completion improved engagement, and reduced confusion and missing data.

Paeārahi discussed that being embedded within a healthcare practice was an essential mediator of pilot success enabling transitions of care, clinical oversight and support when cases moved beyond the paeārahi’s expertise. Participants suggested that in a comprehensive assessment and service pathway, paeārahi could undertake the assessment, pass results on to others within the care team for care planning and then support the wraparound services that were recommended. Paeārahi valued ongoing support of interRAI educators and educators felt the level of support required post-training was like that required by competent interRAI assessors.

Quantitative acceptability, feasibility and healthcare access measures are shown in Table 3.

**Table 3.** Quantitative Acceptability, Feasibility and Healthcare Access Outcomes

Measure	Intervention participants (n = 50)
Acceptability	
Reported confusion or intrusiveness with at least one assessment item	22%
Feasibility	
Recruitment	
Recruitment rate (n = 71 approached)	70.4%
Reasons for non-participation: lack of time, not wanting to participate in research assessment	
Withdrawals	
Completion method	0%
Self-completion	8%
Paeārahi facilitated completion	92%
*Via phone*	*66%*
*In-person*	*26%*
Contact time to complete per assessment (mean [range])	33 minutes (30-60)
Non-contact time per assessment (mean [range])	30 minutes (30-30)
Paeārahi travel time per assessment (mean [range])	7.3 minutes (0-40)
Healthcare access – Referrals	
Primary care physician	28%
interRAI assessment with trained assessor	4%
Practice nurse	6%
Physiotherapist	4%
Health improvement practitioner	4%
Rongoā practitioner^a^	4%
Other^b^	8%

^a^A practitioner trained in Māori traditional healing systems

^b^One referral was made to each of the following professionals or services: pharmacist, hearing test, another paeārahi, routine screening test

### Healthcare Access

Older Māori and provider participants perceived the assessment was useful to improve access to healthcare feeling that paeārahi could take on activities which eased pressure on primary healthcare workforces. Paeārahi reported the assessment added value to their usual assessment and care planning. Paeārahi noted that strong working relationships with general practice staff supported successful implementation and smooth care transitions. Some older Māori said that they were already strong advocates or already had appropriate supports and networks in place for themselves and did not necessarily find the assessment useful.

During the project, paeārahi deepened their knowledge of services that were available to support older Māori at home. This is an important learning as, independent of the CU-SR, increased support for paeārahi to understand the aged care sector could improve well-being. One paeārahi, for example, described the value of talking to a home care support worker to better understand about the referral processes—such as making a direct referral to an occupational therapist instead of waiting for an interRAI assessment.

### Importance of the Paeārahi Role

Paeārahi were essential in the CU-SR process due to their ability to engage, support assessment completion and provide connected wrap-around services. One participant described that the CU-SR was better than the other formal assessments they had been part of although this was related to the paeārahi wrap-around care as opposed to the assessment tool itself.

## Discussion

We demonstrated facilitated completion of CU-SR with older Māori is both feasible and acceptable. Most items were well-received and provided participants with valuable health information. Our model, where paeārahi supported CU-SR completion and related care planning, helped deliver culturally appropriate care for Māori and was seen to improve access and integration across the health system. As populations age and demand on health services grows, , it becomes increasingly important to direct services towards those who will benefit most and to utilise a diverse, connected health workforce.

Others have demonstrated the reliability of CU-SR when used by both trained registered and unregistered health workforces^4^ and have proposed strategies for integrating the tool into primary care service pathways.^18^ Our work extends this by demonstrating acceptability in an Indigenous context and by incorporating care planning and facilitated healthcare access through trained, unregistered health and social care practitioners.

Beyond supporting care planning, we found that the CU-SR can help identify unmet assessment or care needs that might otherwise go unrecognised, while by reducing reliance on trained assessors and helping conserve limited workforce resources. The supported self-assessment process also empowers older Māori to recognise and articulate their own informal or formal support needs. These outcomes align with strategies within the World Health Organizations ‘Framework on integrated, people-centred health services’,^19^ highlighting the critical role of clinical information systems such as interRAI in enabling effective, responsive and equitable health system performance.

Access to aged care services remains inequitable for some groups in NZ, including Māori,^5^ despite known benefits of these services for individuals and society. Navigator roles in other contexts have improved equity of access^9,20^ and our care model shows similar promise in improving access to formal aged care services.

The paeārahi training contributed to this success. Experts in interRAI education, along with health professionals and trained assessors from secondary care in the local health region, delivered the training and remained available to support paeārahi throughout the pilot. Their openness to reciprocal knowledge exchange—recognising that they also learned from paeārahi—fostered mutual trust and strengthened relationships across provider groups. This spirit of knowledge sharing also shaped the wider development of the pilot.

A similar openness to knowledge exchange shaped the development of this pilot. Through co-design with paeārahi, Māori health providers and older Māori, we embedded Māori values and ways of working into the interRAI assessment training, intervention delivery and evaluation. This collaborative process increased the likelihood of acceptability, meaningful engagement and positive impact from the care model.^21^

Building on the successes and challenges identified, post-intervention feedback paeārahi, other health providers and older Māori highlighted areas for improving the model in future iterations. Embedding paeārahi within existing health practices was crucial - a finding supported by other research exploring navigator roles noting that emphasizes the need to establish trust, and develop shared communication methods and systems for effective practice.^22,23^ These elements, including the related infrastructure to manage referral and follow-up pathways, represent key considerations for future research and implementation, especially when exploring the independent completion of the CU-SR for broader population screening.

## Limitations

This study was conducted with older Māori enrolled in one NZ practice and results are not widely generalisable. Scales were not embedded in software during the intervention phase which meant the impact of this knowledge on courses of action taken by paeārahi could not be assessed (these have since been embedded in the NZ iAS). The ADL scale did not demonstrate internal reliability, likely due to lack of data variance in that scale (largely independent study population). The study excluded those known to need interRAI assessment and further study is needed to assess the appropriateness of the use of facilitated CU-SR in more complex individuals.

## Conclusions

Paeārahi-facilitated CU-SR assessment and care planning is a scalable model utilising a culturally appropriate, non-regulated Indigenous health workforce. The incorporation of Indigenous values and workforces should be considered when implementing services in other First Nation populations, particularly when using assessments validated in other populations.

The use of the CU-SR demonstrates the potential to identify unmet need, reduce workforce shortages, and address inequities in aged care access. Future investigations should explore effectiveness and implementation strategies across more diverse healthcare settings.

## Data Availability

Data is not available to other researchers as ethics approval was not granted for others outside the research group to access the data.
